# Civic Participation in Chinese Cyberpolitics: A Grounded Theory Approach of Para-Xylene Projects

**DOI:** 10.3390/ijerph182312458

**Published:** 2021-11-26

**Authors:** Xin Yu, Jinpeng Wang, Yongliang Liu

**Affiliations:** School of Journalism and Communication, Tsinghua University, Beijing 100084, China; wangjp18@mails.tsinghua.edu.cn (J.W.); liuyongliangleo@163.com (Y.L.)

**Keywords:** mediatized politics, civic participation, cyber deliberation, grounded theory, political communication, political logic, mediatization, environmental protection

## Abstract

The internet provides a free and convenient platform for the public to obtain political information and participate in political life. Meanwhile, there occurs fierce confrontation of various values and ideologies, shaping a complicated and changeable field of public opinion. The strategies of civic participation and the generation of public opinion show quite different characteristics in such a mediatized society. This article aimed to study civic participation in Chinese cyberpolitics and to find its patterns and the logic behind it. Due to the natural advantage of the environmental issues in its commonality, the internet events in the last decade related to the PX (para-xylene) project were selected as the research object. This study used grounded theory as the method and conducted a cross-case analysis on the original data captured on Weibo—one of the most popular social media sites in China. Finally, four patterns of civic participation in internet events were found and summarized, as well as the intervention and influence of media logic in different modes. However, it is political logic, rather than media logic, that reveals greater vitality in the civic participation of cyber deliberation. Mediatization does exist but is far from dominance. It has certain significance for the supervision and management of public opinion and the rational and harmonious development of civic participation in public issues.

## 1. Introduction

The internet is an important platform for public political participation. Social media stimulates civic politics participation and interpersonal communication on the internet [1]. The digital platform can support democratic politics and fulfill their role as a public realm [2]. Some researchers believe that netizens change the model of public agenda setting through internet political participation [3]. However, online discussions have not always changed people’s inherent concepts and cognition as we expected. Besides technological environment and institutional factors, knowing how to shape a cooperative political culture is crucial [4]. During the Western environmental movement in the latter half of the 20th century, scientific environmental knowledge has become the consensus of the community as the basis of public debate. However, in China’s environmental protection movement based on social media, scientific knowledge, which is representatively ‘lizhongke’, a Chinese social slang that means rational, neutral and objective, has become the symbolic standard of ‘faction division’, and intellectuals have formed ‘party spirit’ based on subjective prejudice [5].

In recent years, a growing number of environmental conflicts in China has led to a significant challenge for local governments [6]. Due to the importance of environmental protection topics, they have increasingly become an issue of constant concern to Chinese urban residents, and a series of online protests on PX (para-xylene) chemical projects have been staged around the country. ‘Carcinogenicity’, ‘high toxicity’ and ‘causing deformity’ are considered as the dangers of projects and also the ‘background knowledge’ of protests [7]. When refuted by scientific evidence, the actors of the online protest respond with words such as ‘you can try and drink it if no poison’ [8]. Whether in Xiamen, Dalian, Ningbo, Kunming, Chengdu or Maoming, the results of the protest on PX seemed to end with ‘the victory of people’—government officials apologized, and the project was suspended or relocated. Therefore, it is necessary for us to know how the public organized these environmental protection campaigns on the internet and what the patterns were of civic participation in Chinese cyberpolitics.

Previous studies on internet-based environmental protest events mostly started from a single case and analyzed the process mechanism of the event, such as resource mobilization, framework integration and opinion leaders [9,10]. Meanwhile, when PX projects in different cities were trapped in the cycle of ‘stop, suspend or move as long as they protest’, the cognition and views of Chinese internet public opinion on the project are not identical [11]. The leading public opinion of the project has gradually expanded from the ‘carcinogenic’ and ‘highly toxic’ at the early stage to the discussion on its ‘low toxicity’ and ‘safety’ and also from the attribute of PX itself as a chemical to the risk and safety issues of the plants’ site selection. Through the virtual ethnography analysis of the ‘PX entry Battle’ in Maoming, some scholars argue that the internet public opinion has changed from ‘polarization’ to ‘rationalization’ [12]. However, when exploring the relationship between media orientation and public reading behavior in Chinese internet news, some researchers use the selective spiral theory to explain the public opinion cycle of the internet and find that the behavior of public receiving, reading and forwarding news presents the feature of ‘selectivity’ [13]. In such a spiral, the propagation influence of a certain coordinate will be continuously stimulated and amplified due to the different biases of people’s information selection, and even the phenomenon of polarization will occur.

The PX protest movement is one of the most representative serialized social environmental protection campaigns in China in recent years. From 2006 to 2014, there occurred serialized PX protest movements. After 2014, the disputes about PX have vanished. However, the civic cyberpolitics participation patterns during these movements still affect current environmental protection campaigns in China. We believe that the study of several influential PX key cases can better reveal the framework construction of such a topic, the formation of its public opinion and, finally, the mechanism of civic participation in public health. It is of great theoretical and practical significance for us to understand the political, economic and social-cultural symptoms behind it, as well as the position and role of the media in it. More importantly, based on the above findings, we can propose more targeted public health policies.

Scholars hold different views on the evolution path of internet public opinion in a single case, while cross-case studies on how the public opinion of the PX project has evolved over the years are rare. As a research object with rich frameworks and lasting for many years, the PX project has great theoretical construction space. Grounded theory can help us to deeply analyze the logic of Chinese civic cyberpolitics participation in PX protests and summarize their patterns. Based on these patterns, we could also construct Chinese environmental protection movement frameworks.

## 2. Literature Review

As mentioned above, in Chinese internet events related to the PX project, there is a diametrically opposite issue framework. In the early events, the dominant framework of internet public opinion is relatively consistent. It is generally believed that the resistance to the PX project has formed public pressure on the government’s ‘private’ decision, and the subsequent choice of the government to adjust policies is regarded as a victory of the internet struggle and a symbol of civic progress. However, in the several events afterward, though the opinions above still exist, a ‘skepticism’ opinion arises and occupies a considerable degree of the public opinion field. In this climate of public opinion, it is generally believed that the PX project is of ‘low toxicity and low harm’, and the protest and criticism of the PX project are irrational. In other words, instead of promoting adequate discussion and rational advice on public issues, the internet has allowed populism to flourish.

It is obvious that the internet plays a more important role in recent social movements than it used to, and social media offers an essential platform for environmental mobilization [14]. This newest form of media is utilized as an approach to express and fight for specific political appeal, not only in the environmental issue but in all kinds of movements. In addition to China, many social movements erupted in Western countries also show this new characteristic. In the absence of clear demands and solutions, the personalized action framework such as “We are the 99%” spreads rapidly worldwide through social networking sites such as Facebook, Twitter and Tumblr. In addition, the recent movements related to racial issues (#ICantBreathe) or gender issues (#MeToo) have fully proved the internet’s ability of civic participation and social mobilization. It is noticeable that the media has deeply intervened in the process of politics, not only as a utilized means but as a transcendental form that modifies the political mechanism, causing a phenomenon called ‘mediatized politics’. More importantly, there are also frameworks and patterns behind environmental protest communication [15].

However, whether the internet has improved or obstructed the construction of the ‘public sphere’ is rather inexplicit, especially in such a mediatized society. There are two main different views of the internet’s impact—optimism and pessimism—both of which represent a specific analytical approach to the media’s influence on civic deliberation. These two different perspectives lead to diverse results of civic cyberpolitics participation. Under the influence of internet optimism, the public is more likely to participate in political life through deliberation and solve social problems in a peaceful way. Meanwhile, with the effect of internet pessimism, the public prefers engaging in politics through action, which means they may appeal to violence. Through these two opinions, we could analyze the motivation and logic behind civic cyberpolitics participation.

### 2.1. Internet Optimism

From the perspective of technology and politics, internet optimists mostly start from the angle of ‘empowerment’, believing that the internet provides opportunities and platforms for public political participation. Drawing on the previous classification, this kind of network power can be divided into three levels according to ten core dimensions, namely e-enabling, e-engaging and e-empowering [16], which correspond to three kinds of behaviors, namely ‘access to information’, ‘request for advice’ and ‘active participation’ [17], also known as the internet as information resource, communication medium and public sphere [18].

Internet optimism believes that the internet’s potential comes from offering exposure to people who used to be excluded by traditional mass media and political debate through the use of the internet and helping them express their interests [19,20].

The internet can improve the scale and speed of political issues, reduce the cost of knowledge acquisition, promote the development of the public sphere and the occurrence of political discussion [21] and form public opinion and multiple discourse spaces and public opinion environments [22] so as to achieve the prosperity of citizen deliberation and promote the development of deliberative politics [23]. Moreover, at the individual cognitive level, the development of the internet also helps to improve individual political knowledge, political participation willingness and political efficacy [24,25].

The internet enables individuals to express their views more independently, without intermediary control or influence. Online social network platforms have added new channels of political participation, providing additional opinion expression platforms and direct political participation for citizens beyond the formal political system, such as online political forums and citizen journalism. These are new direct political participation ways. Meanwhile, internet application also breaks through the limitations of time and space and expands the opportunities for individuals to express and receive information [26].

In terms of social mobilization and government response, internet optimists also believe that the internet’s technical facilities and its corresponding cyberculture have stimulated the mobilization process of political participation [27], especially for adolescents [28,29]. In addition, the internet use of government also improves its governance efficiency and responsiveness and enhances individual internal efficacy through such a way [30]. Even after expressing deep anxiety about class differentiation and group polarization caused by the internet, Sunstein still believed that, although the effect of group psychology on the network is still common, the healthy and useful information is highlighted and plays the role of public deliberation in some recent internet events [31].

### 2.2. Internet Pessimism

From the perspective of individual cognition, the polarization of internet opinions and the solidification of individual opinions are important footholds for internet pessimists to criticize the optimists [32]. It is difficult for holders of different standpoints to reach a consensus in conflicts [33]. These beliefs all come from the assumption that online public opinion is ‘scattered and diverse’, and individuals will filter information according to their existing cognitive and subjective needs, resulting in the ‘information cocoons effect’ [31]. For example, in a representative sample of 34 major cities, 54.8% of the respondents were ‘unaware’ or ‘very unaware’ of PM2.5 [34].

From the perspective of social structure, researchers believe that the political imbalances caused by the digital divide will not be reduced by the internet but further exacerbated [35]. At the mobilization structure level, unlike internet optimism believing the internet to promote political mobilization, researchers consider information as the basis of both mobilization and de-mobilization, and the latter of which is based on the assumption that individuals may inhibit their social mobility when they perceive widespread ‘ignorance’ of online public opinion.

From the perspective of political economy, the research on the commercialization and control of the internet has also become a powerful weapon for the criticism of internet optimism in recent years. In Times of the Technoculture: From the Information Society to the Virtual Life, Robins and Webster bluntly described the virtual world we live in as an ‘electronic panopticon’ [36]. The authors emphasized the structural violence behind information technology, which on the one hand, inherits the social management and control relations at the macro level and, on the other hand, also shapes daily life and cultural practice at the micro level. They also pointed out that it is capital that manipulates the ‘unipolarized’ network public sphere in China [37].

### 2.3. Disputation Focus and Existing Framework

In summary, the disputation focus of internet optimism and pessimism on civic participation and social mobilization mainly has the following three aspects.

Firstly, is the scale of political participation enlarged or shrunken? Internet optimism believes that the internet reduces the flowing cost of political knowledge and issues, breaks the limitation of time and space [38] and expands the channels of political participation. Nevertheless, internet pessimism argues that the expansion of political information liquidity does not equal the enlargement of civic participation. With a phenomenon such as the digital divide, the inequality will increase and even block the approaches for citizens to participate in politics.

Secondly, is the public opinion integrated or polarized? Internet optimism believes that as a ‘public deliberation platform lasts for long time’ [39], the internet encourages the reflexivity and argumentation of individual statements and thus is conducive to civic deliberation [40]. On the contrary, internet pessimism believes that the internet cannot promote the communication and criticism between different ideas but leads to the divergence of opinions, resulting in the homogeneity and polarization of public opinion due to the individuals’ dependence on their inherent tendency instead of facts [41].

Thirdly, is the individual mobilized or de-mobilized? Internet optimism believes that the internet promotes the political self-efficacy of individual netizens, fosters political participation and facilitates the ways and channels of political mobilization. Meanwhile, pessimists argue that the initiative of individual netizens will lead to political indifference and unwillingness to express views or opinions on public issues because they perceive the climate of ‘ignorance’ or ‘opposition’, and therefore, the political actions are inhibited.

This article studies the patterns and logic of civic participation in internet events from the perspective of an internet public opinion framework. Because of the natural feature of the environmental issues in its wide-range commonality, almost every citizen has the right to judge or comment on the public policies for environmental protection. The internet undoubtedly provides the appropriate platform for the discussion, and it so happens that citizens adopt this mediatized method to fight for their rights, especially when it comes to their personal interests. However, as a specific territory, environmental protection has its knowledge barrier, which makes it professional, and thus, disputation occurs usually in the public discussion and argument.

However, before we look deeper into the specific topic of internet events, it is better to examine the existing framework in similar studies on internet events. It is found that the framework mainly has the following four types of definitions.

The first category, which is of the macro level, has summarized and classified cultural framework resources from the online and offline group events in China. For example, there is protest according to law [42], protest according to the situation [43], protest with death [44], individualistic resistance [45], the ‘making it severe to earn attention’ (NAO-DA) resistance [46], performing resistance [47] and other types.

The second category, which is of the micro level, is the direct presentation of the specific framework in the process of event development. For example, there are ‘Shield in hand, helmet on head/law enforcement, civilized law enforcement/maintain order’ [48] and a series of frameworks presented in the study of Wukan events [49], whose specific content is referred to in Table 1.

The third category, which is of the middle level, is the further induction of the specific framework but still in relation to the topic. For example, there is beggar-thy-neighbor/policy advocacy [50], pollution level/public health/economic loss [51] and disaster description/problem interpretation/accident investigation [52].

The fourth category, different from the content framework above, focuses on the involvement of different subjects in the development of events. For example, there are expert positions/elite consciousness [53] and central media involvement/opinion leader involvement [54].

In general, there are many studies on the framework analysis of PX project events, but there is still a lack of certain typology and logic, especially a lack of literature that combines the content framework and the subjective framework. This article, through the method of grounded theory, focuses on the combination of the medium level of the content framework and the subjective framework to form a relatively complete framework type of similar internet events. We discuss how the combination of the content framework and the framework of the participating subjects is carried out in different civic participation patterns, as well as how the media connection plays a role in different patterns. Finally, this study then interprets the results and discusses the theoretical significance behind them.

## 3. Research Process

### 3.1. Methodology: Grounded Theory

The grounded theory is the methodology of theoretical construction through continuous comparison and inductive analysis based on rich and diverse empirical materials. Generally speaking, researchers do not have very fixed theoretical assumptions before conducting grounded theory analysis but start directly from phenomenon observation and empirical materials and form theoretical generalizations through a series of normative research processes from bottom to top. Some scholars have used the research method of grounded theory to explore the driving factors and generation mechanism of trust transfer in network events. Through open, axial and selective coding [55], researchers have abstracted the theoretical concepts from news databases and WeChat public accounts and thus explained the model and trend of trust transmission in network events. To conduct exploratory research in the absence of empirical research, researchers usually develop the theory based on data facts.

As mentioned above, the research on internet public events, especially the different frameworks formed in different internet events, has high contingency and instability. If we mechanically refer to previous study results and literature summary, it is likely to lose rich discourse frameworks and a series of internal patterns behind them. Therefore, this study adopted the method of grounded theory, trying to present the rich practice and performance of the research object more completely.

### 3.2. Data Collection and Sampling Method

Searching with ‘PX’ as the keyword, the original microblog data of Sina Weibo from 2 September 2009 to 19 April 2014 were captured through the Web crawler program and the API interface provided by Sina Weibo—one of the most popular social media sites in China. A total of 456,993 original microblogs were obtained, and the capture time was from 18 to 23 October 2014. Because this research hoped to study the microblog text that is to some extent typical, the sampling method adopted was mainly purposive sampling, specifically the original microblog text with a certain number of forwarding. Meanwhile, due to a large number of irrelevant text entries when searching by ‘PX’, the authors carried out artificial screening after sorting the microblog text by the number of forwarding from high to low. Finally, the authors received 181 original microblog texts related to the PX project, whose number of forwarding was more than 100. They mainly covered the events that occurred in Chinese cities, including Dalian in 2011, Ningbo in 2012, Kunming in 2013 and Maoming in 2014.

### 3.3. Encoding Process and Initial Results

In this study, the encoding process was carried out in accordance with the steps of grounded theory. (1) The first step is open coding, in which the selected microblog samples are coded freely. The relevant definitions of the concepts are summarized and refined according to the text content. In this process, the literature involving related concepts is reviewed to make the definition more accurate. (2) The second step is axial coding, in which open coding is aggregated according to the content correlation. With the assistance of software, the relationships between free codes are identified to find the theoretical significance behind the open coding of mutual relationships, which includes not only the tree-shaped subordinate relationship but also the linear correlation and organic combination. Then preliminary axial coding is constructed and integrated with the results of open coding to form a theoretical model. (3) Finally, the third step is to test the validity and data saturation of the formed theoretical model. NVivo 11 was used as the coding and analysis tool in this study.

#### 3.3.1. Open Coding

According to the above steps, at the stage of open coding, the researchers conducted a word-by-word review and analysis of the content of microblog texts and summarized the original content. In the initial stage, 39 free nodes were abstracted from the content. The details of the free nodes and corresponding encoded text content (referred to as ‘references’ in NVivo 11) are shown in Table 2.

#### 3.3.2. Axial Coding

Based on the repeated comparative analysis of open coding, we formed a number of axis nodes to understand the text more structurally. In the process of axial coding, researchers generally classify the axis nodes by six main categories: problem definition, causal explanation, moral judgment, countermeasures and suggestions, media connection and rhetorical methods.

In problem definition, causal interpretation, moral judgment and countermeasures and suggestions, the researchers distinguished whether it was aimed at the PX project itself or the PX protest events. Media connection refers to quotation from the media beyond the expression of the content, in which ‘media content’ mainly includes domestic protest cases and foreign movement cases, which is the extension of the event in time and space, while ‘media subject’ includes traditional media and opinion leaders. Rhetorical methods, from the perspective of narrative analysis of the text form, mainly include analogy. The relationship between axial coding and open coding, and the conceptual definition of free nodes, is shown in Table 3.

#### 3.3.3. Saturation Test

In the process of qualitative research, the scope of the sample is continuously expanded until the new concept or relationship cannot be extracted from the new sample, which means that the research process has reached ‘data saturation’. After determining 181 sample texts, we first randomly used two-thirds of the sample (a total of 121 texts) for open coding and axial coding and constructed the relational structure and theoretical model of response. Then the remaining one-third of the sample (a total of 60 texts) was used for the data saturation test. The test results show that the constructed theory has sufficient and complete categories, and no new important categories or relationships are found when coding and analyzing the remaining one-third of texts. Therefore, it can be considered that we reached data saturation in theoretical construction.

## 4. Research Findings: Four Patterns of Civic Participation

**Figure 1:**
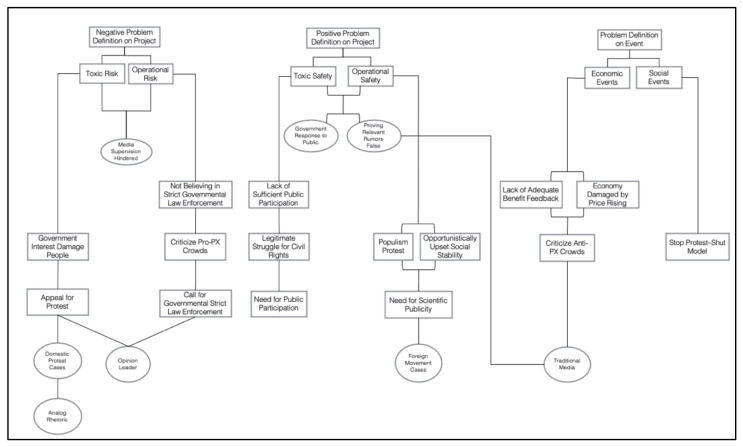
Main category relationship structure diagram.

By comparing and combining free nodes with each other, this study attempted to construct several patterns of civic participation in internet events. Starting from different problem definitions of projects or events, the framework structures of relevant causal interpretation, moral evaluation, treatment recommendation and corresponding media association and rhetorical method were also presented. After generalization and summarization, this study concluded four theoretical patterns, namely risk resistance pattern (toxic risk), citizen deliberation pattern (operational risk, toxic safety, social events), scientific communication pattern (operational safety) and economic rationality pattern (economic events). For specific structural details of variables and its categories, we make them more clear and present them in Figure 1 and Table 4.

### 4.1. Risk Resistance Pattern

Risk resistance pattern refers to the mode that the public perceives a high risk exists in the project and thus takes actions to achieve the purpose of appealing for protest action and stopping the project from being put into production. In this process, they are not concerned with the significant causal interpretation behind the project or event but expect to mobilize others to participate in the protest action through critical moral evaluation of the government. In addition, they would also use the previous cases of protests in other domestic cities or regions as references, give them legitimacy as a template for the success of the protest and prompt the possibility of the same success this time. In the process of mobilization, the risk resistance pattern would also expand its influence by connecting opinion leaders in other ways, hoping to attract more attention. It is a pattern that expects to use the internet for empowerment.

In this pattern, participants focus on the project definition as toxic risk because it is more negative compared to the relatively moderate operational risk, and thus it can frame actions that trigger others. It is the same when using media supervision hindered as the progress of event procedure in order to unite more allies such as the media. The analogy rhetoric is also used to strengthen the emotions of expression, aiming to form an opposite situation of the government versus enterprise, as well as the public versus media, and strengthen the moral evaluation of government interest damage people.

### 4.2. Citizen Deliberation Pattern

Compared with the appeal for the protest movement, which is the foothold of the risk resistance pattern, the citizen deliberation pattern does not concern the abolition disputation of specific projects but focuses on the analysis of the causal interpretation of events and their future treatment. In this pattern, according to the different problem definitions of the project or event, the three cognitions of operational risk, toxic safety and social events have developed different citizen deliberation contents.

In the category of perceiving the project as operation risk, although it belongs to the negative definition of the project as well, it is different from the definition of toxic risk. The definition of operational risk has actually recognized the attribute characteristics of PX’s ‘low toxicity and low harm’ identified by scientists, and thus the focus of the discussion is on the project location and management level. It is considered that not believing in strict government law enforcement is the causal interpretation for large-scale protests. Correspondingly, in the aspect of treatment recommendation, it is also considered that the key to the survival and management of future PX projects lies in whether the government can strictly enforce the law, standardize the operation of the project and avoid operational risks. Relatively speaking, since the scope of operational risk is still a negative definition of the project, it also connects opinion leaders to enhance their influence and attention. It is, however, worth noting that, unlike the dichotomy between government/enterprise and public/media, which is emphasized in the risk resistance pattern, the operational risk in the citizen deliberation model is relatively critical, but it still agrees with the positive role that the government should play in it. Rather than the opposition between the government and the public, it is more likely that the government represents the public and regulates enterprises according to law, which emphasizes the relationship between the government and enterprises.

The second specific category of the citizen deliberation pattern is based on the cognition of toxic safety on the project. In such a context, the definition of projects is largely positive, with the focus on explaining why ‘relatively safe projects are subject to public protest’. Through the analysis of open coding, this category believes that the main causal interpretation is the lack of public participation, and the corresponding moral evaluation of protest events also believes that this is a legitimate struggle for civil rights, and therefore, the final treatment recommendation is a need for public participation. In the coding of the progress of event procedure, the category of toxic safety also includes the recognition of government response to public and the persistence of proving related rumors false. In general, on the fact level, this category upholds the attitude of rational science and criticizes the views contrary to the facts. On the value level, it criticizes the government’s neglect of civic participation in the process of political decision making. In this category, media association mainly refers to the relevant reports of traditional media when related rumors are falsified, and traditional media plays a fundamental role in providing accurate facts in the era of social media.

Another category of the citizen deliberation pattern is identifying the PX event as social events. This category has relatively not much relationship structure, mainly in the moral evaluation level that should stop the state of ‘protest–shut’ reaction. In general, it is consistent with the attitude tendency of the other two categories of operational risk and toxic safety. It is believed that the government should have more improvement in similar events in the future, and it is expected to reach a consensus through public discussion and civic deliberation.

### 4.3. Scientific Communication Pattern

Scientific communication pattern refers to the mode that the public considers the project safe and holds critical attitudes to a series of protests. Similar to the category of toxic safety in the citizen deliberation pattern, this pattern also chooses to present the facts of the event’s progress such as government response to public and proving relevant rumors false in order to set the record straight. However, different from the discussion on the complex causal interpretations in the citizen deliberation pattern, the scientific communication pattern does not make a relatively detailed causal explanation, though it is mostly based on the principle of ‘fact first’. Instead, it focuses on criticizing a series of protest events at the level of moral evaluation, mainly believing that it is a populism protest and will opportunistically upset social stability. In the future treatment recommendation, the scientific communication pattern believes that popular science publicity should be strengthened, which is also in line with the criticism of the irrational characteristics of populism. Meanwhile, the science communication pattern would quote foreign well-operated cases to refute the view of toxic risk and operational risk, claiming that the PX project essentially does not have toxic or operational risk.

### 4.4. Economic Rationality Pattern

Compared with the citizen deliberation pattern, the economic rationality pattern mainly emphasizes the gains and losses of PX events in economics, rather than the value disputation of political or social public affairs, though both of them are relatively isolated from specific events and cases to be discussed. If the series of events are characterized as economic events, then the lack of adequate benefit feedback is considered to be the cause of the protests, while an economy damaged by price rising is the result of the protests, both based on the framework of economic gains and losses. As to the moral evaluation, the economic rationality pattern would criticize anti-PX crowds, and the specific logic is that the people who oppose PX lack rationality. Civic participation will, on the contrary, lead to a supply shortage of PX products in China, increase the dependence on foreign PX products and cause damage to the development of related industries. In terms of media association, the economic rationality pattern is mainly connected to the reports of traditional media. However, different from the falsification of rumors in the citizen deliberation pattern, the traditional media here mainly plays the role of reporting economic news, such as the PX import and export situation, as well as its price. Either way, the basic role of the traditional media in the two patterns is to provide the exact facts.

## 5. Conclusions and Discussion

### 5.1. Action Approach of Mediatized Politics

Among the four patterns of civic participation in internet events—risk resistance, citizen deliberation, scientific communication and economic rationality—the risk resistance pattern embodies more action characteristics, specifically reflected in the lack of two types of frameworks—‘causal interpretation’ and ‘moral evaluation on event’. It mainly presents the government interest damage people in the moral evaluation of the project. The approach of the risk resistance pattern is relatively concentrated and carried out around the project itself. It defines the project itself as toxic risk, skips the causal interpretation and makes the moral evaluation of the project as government interest damage people, and eventually, the treatment recommendation appeal for protest is proposed.

Arendt pointed out in The Human Condition that, compared to the necessary labor and useful work, action is a realization of human self-display and also a necessary condition for people to obtain a sense of reality [56]. Action is not purposeful, and it is exactly the purposelessness that leads to the uncontrollability of group action. From the perspective of psychological analysis, it is also found that the significance of action in the internet space may not lie in objectivity and rationality but in the need for self-display or the adjustment of ‘disadvantaged psychological status’ [57].

In the ‘action’ approach of the risk resistance pattern, the role of the media in it mainly reflects the function of ‘empowerment’. Our study found that, in the pattern of risk resistance, the mediatized methods are mostly used to fight, mainly including citing the domestic protest cases to prove its legitimacy, connecting the opinion leader to realize the network communication and cross-platform diffusion of information and using the method of analogy rhetoric to strengthen the emotion and enhance the possibility of causing public emotional resonance.

### 5.2. Deliberation Approach of Mediatized Politics

The citizen deliberation pattern, scientific communication pattern and economic rationality pattern mainly reflect the deliberation approach of civic participation in internet events. Such deliberation can be political or based on scientific knowledge or economic rationality. By analyzing the patterns of such civic participation in internet events, it was found that most of them conduct the causal interpretation of the PX event itself. For example, in the citizen deliberation pattern, it is believed that those who regard the project as operational risk will attribute the cause of frequent protests to the disbelieving in strict governmental law enforcement of the public, and those who regard the project as toxic safety will attribute it to the lack of adequate public participation, which is based on a positive attitude to the problem definition. Meanwhile, in the economic rationality pattern, the causal interpretation for the occurrence of the series of events is considered in a more in-depth way, which believes that it may be due to the lack of adequate benefit feedback and meanwhile cause the economy damaged by price rising. This pattern is the only framework discussing the consequences caused by the PX event.

There are also great differences between the deliberation approach and the action approach in the framework types of moral evaluation. Under the action approach represented by the risk resistance pattern, civic participation essentially only makes moral evaluation of the project itself; meanwhile, under the deliberation approach, civic participation makes moral evaluation more of a series of PX internet events. In the citizen deliberation model, for example, there is criticize pro-PX crowds, legitimate struggle for civil rights, stop protest–shut model and other event moral evaluation frameworks. In the scientific communication model, there is a view that the series of protests are populism protests and there is a possibility of opportunistically upset social stability. Meanwhile, the economic rationality pattern is contrary to the moral evaluation framework of criticizing pro-PX crowds under the citizen deliberation pattern but shows a framework of criticizing anti-PX crowds. However, although the moral evaluation frameworks of these two patterns are completely contrary, their common feature is that they tend to judge events morally rather than be confined to the project itself.

In the part of treatment recommendation, the three patterns under the approach of deliberation are also putting forward some policy advocacy, such as strict government law enforcement, need for public participation, need for scientific publicity and so on. Compared with the appeal for protest, which is a beggar-thy-neighbor strategy under the approach of action, the deliberation approach reflects stronger publicity.

Through comprehensive analysis of the difference between the deliberation approach and the action approach, it can be found that the deliberation approach would be more of the commonality, which is beyond the specific project and interests, exactly as conflict between ‘right and wrong’ instead of ‘interest’. The deliberation approach tends to make causal interpretation and moral evaluation of the series events, rather than specifically of the PX project itself. Even in moral evaluation, it is common to stand in the public interest and the future long-term perspective, whether the judgment is supportive or critical. In the part of treatment recommendation, the deliberation approach also makes policy recommendations from the perspective of policy advocacy, which reflects the characteristics of civic deliberation from ‘beggar-thy-neighbor’ to ‘policy advocacy’. On the contrary, the risk resistance pattern under the action approach shows the mobilizing action characteristics are more concentrated on the project itself, and in policy recommendations, it also adopts the suggestions that are ‘beggar-thy-neighbor’, completely different from the deliberation approach.

As for the media association, although the connection to opinion leaders also exists, the main media association is traditional media and foreign movement cases in most patterns of the deliberation approach. Traditional media mainly provides factual information, especially in the process of refuting rumors; meanwhile, foreign movement cases are cited to discuss the safety distance of the PX project built around the city, which is different from the reference of a domestic protest case in the risk resistance pattern to prove the legitimacy of their own actions.

### 5.3. Discussion: The Role of Media

So far, through the grounded theory method and the cross-case analysis, this study found four patterns of civic participation in internet events and, afterward, came up with two different approaches of mediatized politics—‘action’ and ‘deliberation’.

Regarding the role that the media played in the two approaches, it is significant that the media in the action approach is ‘empowerment’, while it is a ‘platform’ in the process of mediatized politics in the deliberation approach. In the action approach, the public would connect to opinion leaders so as to diffuse the information and extend the influence. Domestic protest case is also cited to provide legitimacy, calling for citizens’ action. In the deliberation approach, the public mainly connects to traditional media for factual information and cites foreign movement cases for relevant mature experience, calling for rational public deliberation. Significantly, this mediatized behavior of the deliberation approach is more for the discussion of the deliberation level, rather than the mobilization of the action level.

The different characters of the media in the ‘action’ and ‘deliberation’ approaches also suggest different orientations of mediatized politics. As was summarized in the literature review, there are three main aspects of the disputation between optimism and pessimism on civic participation and social mobilization. The scale of political participation is hard to determine because of the limitation of research methodology—the grounded theory is more qualitative than quantitative.

However, the discussion between the other two arguments can be found in our approaches. Under the action approach, the risk resistance pattern evades the casual interpretation which is of relative rationality and, instead, stresses the moral judgment to encourage taking action on the protest, resulting in the divergence and polarization of public opinion. Meanwhile, the action approach strongly embodies the mobilization effect as well, promoting civic participation by appealing for political action.

Under the deliberation approach, the patterns appeal for rational civic deliberation based on political, scientific or economic fundamentals. More causal interpretations are adopted, and the evaluations are concentrated on the normal circumstances instead of specific projects or protests. Therefore, the media of the deliberation approach serves as a ‘platform’, or a ‘public sphere’, and facilitates the integration of public opinion. As there occurred more than one opinion in the problem definition, it draws the conclusion that the deliberation approach is conducive to extending civic participation, at least in the diversification dimension. So far, no result in this article supports the ‘de-mobilized’ hypothesis.

Further, the ‘action’ approach explicitly embodies the mediatized politics and the leading role of media logic—the attention is so important that such mediatized methods are utilized to draw attention and make the protest salient. However, contrary to the existing recognition, even in a mediatized cyberspace, the ‘deliberation’ approach, or civic deliberation, still follows the basic rules of political logic, which takes the commonality first and calls for public rationality. The media under the deliberation approach is aimed at developing the commonality, instead of drawing attention. It hints at the low degree of mediatization in Chinese cyberpolitics, though both approaches consist of the media as a realization method of political purpose.

## Figures and Tables

**Table 1:** Framework names and types in existing studies.

Issue Types	Framework
Unspecified	Protest according to law [42]; Protest according to the situation [43]; Protest with death [44]; Individualistic resistance [45]; ‘NAO-DA’ resistance [46]; Performing resistance [47]
Public Security Conflict	Shield in hand, helmet on head/law enforcement, civilized law enforcement/maintain order [48]
Environmental Protection	Beggar-thy-neighbor/policy advocacy [50] Pollution level/public health/economic loss [51] Disaster description/problem interpretation/accident investigation [52]
Land Evictions	Natural justice/gender equality/safeguarding rights and interests/supporting the Communist Party/abnormal petition/riots/contradictions among the people/peaceful protests/pole-breaking uprising/new peasant movement/media helps protect rights/overseas forces incited/officials should reflect/democracy [49] Socialism/collectivism/central media involvement/opinion leader involvement [54]

**Table 2:** Reference point examples of open coding.

Free Nodes	Statement Text Examples (References)
Toxic Safety	Various animal experiments have shown that PX has no reproductive toxicity, teratogenicity or genetic toxicity
Toxic Risk	The cigarette is a kingd of drug and you can smoke every day... if the students eat PX every day... then we will believe the PX has low poison
Operational Safety	I have visited a PX factory in South Korea. The factory is only a few hundred meters away from residential buildings
Operational Risk	Now that PX is safe, do I have to accept it being poked right under my nose? When choosing a location for a PX project, will you die if it is far away from the city? Does it have to be within 5 km of a city?
Economic Events	[Life cannot live without PX] PX, paraxylene, is a colorless and transparent liquid at room temperature. It is an important basic chemical raw material with a wide range of uses.
Media Events	The technical term became the buzzword: pm2.5, melamine. thallium. H7N9, PX.
Social Events	The biggest mistake made by scientists is that they do not realize that PX, as a project, is no longer a purely scientific problem, but a social one
Dam Falls in Dalian	The breakwater of Dalian’s 700,000-ton PX project broke, which may spark a new round of PX fears
Kunming Not Produce PX	Kunming government: the refinery project does not produce PX products
Media Supervision Hindered	The chairman of the Dalian PX project was reported to have said that putting journalists in the factory would kill employees
Proving Relevant Rumors False	People’s Daily: “It’s a rumor that the tanks came to town during the PX incident in Maoming.”
Government Response to Public	The Dalian municipal government held an emergency meeting on the night of September 9, putting the relocation of Fujia Dahua PX project on the agenda of addressing Dalian’s environmental risks. It proposed to conduct a comprehensive investigation and assessment of the safety situation of Fujia Dahua PX project, give a scientific and responsible explanation, demonstrate the relocation problem of the PX project, and propose a plan as soon as possible
Tsinghua Entry Defense	PX Entry Defense: Students at Tsinghua University defend PX entry day and night
Government Improve Environmental Governance	Only conduct the PX project can the small factories be closed. Xiamen has moved PX to Zhangzhou. The government has no way to close down any of the original chemical plants. Without that money, even the bus subsidies are gone. It is easy to find that it is cost-efficient to build a big factory rather than a small one with the same regulation.
Lack of Adequate Benefit Feedback	They believe that they cannot get substantial benefits, the quality of life will decline, the interest regurgitation will be insufficient, and the risk will be borne by themselves
Lack of Sufficient Public Participation	The government does not pay enough attention to the public participation in public decision-making, which will eventually take the consequences.
Economy Unharmed for Market Mechanism	Some people say that China must go ahead with the PX project, otherwise the PX will be monopolized by foreign countries and the price will rise. To be honest, I really cannot agree with this view. How can a foreign country monopolize the PX market? If Japan’s PX is expensive, we can buy them from South Korea; If South Korea’s PX is expensive, we can also buy them from the United States. There are so many countries in the world, and we could finally find the cheapest.
Economy Damaged by Price Rising	The price increase of basic chemical materials is not very high, but in recent years, the price of PX has soared from 8000 to nearly 20,000. The United States, the largest producer of PX, and Japan, the largest exporter of PX, are very happy with this result.
Not Believing in Strict Governmental Law Enforcement	Do not trust the government to strictly regulate and enforce the law
Government Interest Damage People	Dalian wants interests, not people’s lives
Criticize Pro-PX Crowds	For those brainless who support PX, I suggest that you eat 1 g of PX every day.
Criticize Anti-PX Crowds	PX project is now a rat crossing the street? They take to the streets to protest without even knowing what PX is. What’s the difference between them and Taiwan’s anti-service trade students.
Populism Protest	Protests by Xiamen residents in 2007 evicted a PX project in the city, which set a bad precedent for the public’s irrational rejection of heavy chemical projects in China
Legitimate Struggle for Civil Rights	Significant results have been achieved in political consultation through microblogs
Public Intellectuals Deceive Public	The public intellectuals do not want to charge, but choose to fool the common people
Opportunistically Upset Social Stability	It also incited street demonstrations, which seriously affected the work and life order of the Maoming people. It was everyone’s responsibility to maintain social stability in Maoming
Stop Protest–Shut Model	The vicious circle of Protest-Shut of PX projects must end
Need for Scientific Publicity	Only by sticking to the bottom line of science can we avoid the PX disturbance
Need for Public Participation	The core of the event is scientific assessment and communication, of which popular science is only one part
Call for Governmental Strict Law Enforcement	We should supervise the government to rectify existing chemical enterprises, and close down all those whose emissions and environmental assessment fail to meet the requirements, gradually reduce the scale and pollution of the chemical industry, and return Ningbo to a pure land
Ask for Governmental Lead	[Reflection on PX disturbance] The establishment of the mechanism of “government-led + public participation + professional judgment” cannot be achieved overnight
Appeal for Protest	Kunming car owners, I hope you can actively participate in this. On the back of your car, please put up this slogan: love my spring city, refuse PX project!
Supervise the Shutdown of Project	The next thing we should do: First, we should supervise the discontinuation of the PX project
Handle Prior-period Investment	How to stop the huge investment of “pre-preparation”
Foreign Movement Cases	Even “garden city states” like Singapore have PX projects, and PX factories in Japan and South Korea are in full bloom
Domestic Protest Cases	Why not learn the lesson of Xiamen’s PX project, which prompted citizens to “walk” and to stop the project
Traditional Media	[People’s Daily confirms: “15 dead, 300 injured” in Maoming anti-PX demonstration is a rumor] In response to a purported photograph of tanks and armored vehicles passing under a bridge on Maoming Avenue and driving on the street, the reporter asked many Maoming residents, none of whom had seen a tank. After verification, the photo is a few years ago a troop training march on the road. In addition, two people were injured in the incident and no one was killed.
Opinion Leader	Girls, please take off your masks because they are made of PX. Please take off your clothes and your bag because they are made of PX. You can be naïve, but not ignorant? @Dan Xiang @Wu Fatian @Du Jianguo Microblog
Analog Rhetoric	It’s scarier than bullet trains! Worse than Fukushima! More hateful than the Ministry of Railways! More hateful than the Japanese invasion!

**Table 3:** The definition of axis nodes and free nodes in axial coding.

Axis Nodes	Free Nodes	Conceptualization
Positive Problem Definition on Project	Toxic Safety	Considering the project itself to be less toxic and less harmful
	Operational Safety	Considering the factory to have advanced technologies, which is safe and solid
Negative Problem Definition on Project	Toxic Risk	Considering the project itself to be highly toxic and harmful, causing the pollution
	Operational Risk	Considering the factory to be placed at a wrong place, in which accidents are easily caused
Problem Definition on Event	Economic Events	Define the events from the perspective of economics and business
	Media Events	Define the events from the perspective of media and culture
	Social Events	Define the events from the perspective of society and governance
Progress of Event Procedure	Dam Falls in Dalian	The breakwater in Dalian broke, rising the risk of PX leak
	Kunming Not Produce PX	Kunming refinery project does not produce PX products
	Media Supervision Hindered	Media to report the event progress but hindered by government and enterprise
	Proving Relevant Rumors False	Refute the relevant rumors
	Government Response to Public	Government hold press conference to respond to public opinion
	Tsinghua Entry Defense	Tsinghua students launched a word entry defense in Baidu encyclopedia
Causal Interpretation on Project	Government Improve Environmental Governance	Because of the advanced technology and scientific management of PX projects, small polluting factories can be forced to shut down to improve the environment without compromising economic interests
Causal Interpretation on Event	Lack of Adequate Benefit Feedback	Lack of financial compensation for residents around the PX project triggered protests
	Lack of Sufficient Public Participation	Lack of public participation in the introduction of PX projects and the EIA process triggered protests
	Not Believing in Strict Governmental Law Enforcement	Protests were triggered by distrust of the government’s ability to enforce strict laws on PX plants
	Economy Damaged by Price Rising	As PX ‘stops as long as protest’, supply is lower than demand, resulting in China’s large import of PX products and price increases
	Economy Unharmed for Market Mechanism	Under economic globalization, PX products have their market supply and demand adjustment mechanism so they will not damage economic development
Moral Evaluation on Project	Government Interest Damage People	The introduction of the PX project is for government officials to harm people’s lives and health for performance and promotion needs
Moral Evaluation on Event	Criticize Pro-PX Crowds	People who support PX construction are foolish, ignorant, conscienceless and deserve to be criticized
	Criticize Anti-PX Crowds	People who oppose PX construction are foolish, ignorant, irrational and deserve to be criticized
	Populism Protest	Viewing protests as irrational, the idea of some dictated government decisions
	Legitimate Struggle for Civil Rights	Viewing protests as rational and progressive actions to promote transparency in government decision making
	Public Intellectuals Deceive Public	Viewing protests as public intellectuals’ fooling behavior for various reasons, deliberately deceiving the public
	Opportunistically Upset Social Stability	Viewing protests as an opportunity for some to disrupt social stability
	Stop Protest–Shut Model	Representatives of all sectors of society need to discuss together to stop the vicious circle of ‘stop as long as protest’
Treatment Recommendation on Project	Need for Scientific Publicity	Thinking the way to solve the dilemma is to strengthen popular science publicity
	Need for Public Participation	Thinking the way to solve the dilemma is to strengthen public participation
	Call for Governmental Strict Law Enforcement	Thinking the way to solve the dilemma is to strengthen government law enforcement
	Ask for Governmental Lead	Thinking the way to solve the dilemma is to persist the lead of government
	Appeal for Protest	Calling on the public to take actions to carry out various protests
	Supervise the Shutdown of Project	Calling for public action to supervise the discontinuation of the project
	Handle Prior-period Investment	Economic input has been made in the early stages of the project, and if it stops, how these inputs should be properly handled requires institutional norms
Media Association	Foreign Movement Cases	Cite good operation cases of foreign cities
	Domestic Protest Cases	Cite protest cases of other domestic cities
	Traditional Media	Cite relevant reports of traditional media
	Opinion Leader	Quote(‘@’) from microblog opinion leaders
Rhetorical Method	Analog Rhetoric	Adopt the analog rhetoric method

**Table 4:** The structure table of main category relations.

	Axis Nodes	Risk Resistance	Citizen Deliberation			Scientific Communication	Economic Rationality
Patterns							
Problem Definition on Project/Event		toxic risk	operational risk	toxic safety	social events	operational safety	economic events
Progress of Event Procedure		media supervision hindered	media supervision hindered	government response to public; proving relevant rumors false	/	government response to public; proving relevant rumors false	/
Causal Interpretation on Event		/	not believing in strict governmental law enforcement	lack of sufficient public participation	/	/	lack of adequate benefit feedback; economy damaged by price rising
Moral Evaluation on Project		government interest damage people	/	/	/	/	/
Moral Evaluation on Event		/	criticize pro-PX crowds	legitimate struggle for civil rights	stop protest–shut model	populism protest; opportunistically upset social stability	criticize anti-PX crowds
Treatment Recommendation on Project		appeal for protest	call for governmental strict law enforcement	need for public participation	/	need for scientific publicity	/
Media Association		domestic protest cases; opinion leader	opinion leader	traditional media	/	foreign movement cases	traditional media
Rhetoric Method		analog rhetoric	/	/	/	/	/

## Data Availability

The data presented in this study were extracted through the Web crawler program and the API interface provided by Sina Weibo.
